# Assessment of coronary artery disease using 3.0T magnetic resonance coronary angiography: a national multicenter trial

**DOI:** 10.1186/1532-429X-15-S1-E5

**Published:** 2013-01-30

**Authors:** Qi Yang, Kuncheng Li, Bin Sun, Hong Yun, Lijun Tang, Shurong Li, Zhenbin Cao, Junling Xu, Mengqi Wei, Lixin Jin

**Affiliations:** 1 Radiology Department, Xuanwu Hospital, Capital Medical University, Beijing, China; 2 Radiology Department, Fujian Union Hospital, Fujian Medical University, Fuzhou, China; 3 Radiology Department, Zhongshan hospital, Fudan University, Shanghai, China; 4 Radiology Department, Jiangsu Province Hospital, Nanjing Medical University, Nanjing, China; 5 Radiology Department, Henan Provincial Hospital, Zhengzhou, China; 6 Radiology Department, First Affiliated Hospital, Sun Yat-Sen University, Guangzhou, China; 7 Radiology Department, Xijing Hospital, Fourth Military Medical University, Xian, China; 8 Radiology Department, Wuhan Union Hospital, Huazhong University of Science, Wuhan, China; 9 Siemens Healthcare, MR Collaboration NE Asia, Shanghai, China

## Background

3.0T contrast enhanced whole-heart coronary magnetic resonance angiography (MRA) is a promising method for noninvasive, radiation-free detection and exclusion of obstructive coronary artery disease (CAD); however, the accuracy of this approach has not been determined in a multicenter trial.

## Methods

An ECG-triggered, navigator-gated, inversion-recovery prepared, segmented gradient-echo sequence was used for image acquisition in 272 patients with suspected CAD at 8 hospitals. The accuracy of coronary MRA for detecting a 50% diameter reduction was determined using X-ray coronary angiography as the reference method. Using an intention-to-diagnose approach, all coronary arteries were included for the evaluation regardless of the image quality of coronary MRA to avoid overestimation of the diagnostic accuracy. Clinical Trial Registration—URL: http://clinicaltrials.gov. Unique identifier: NCT01024478.

## Results

Acquisition of coronary MRA was successfully completed in 235 of 272 (86%) patients with average imaging time of 9.5±1.6 minutes. The areas under the receiver-operator characteristic curve from MRA images according to vessel- and patient-based analyses were 0.90 (95% confidence interval [CI]: 0.88 to 0.95) and 0.88 (95% CI: 0.83 to 0.93), respectively. The sensitivity and specificity of MRA on per-patient basis were 91% and 80%, respectively.

**Figure 1:**
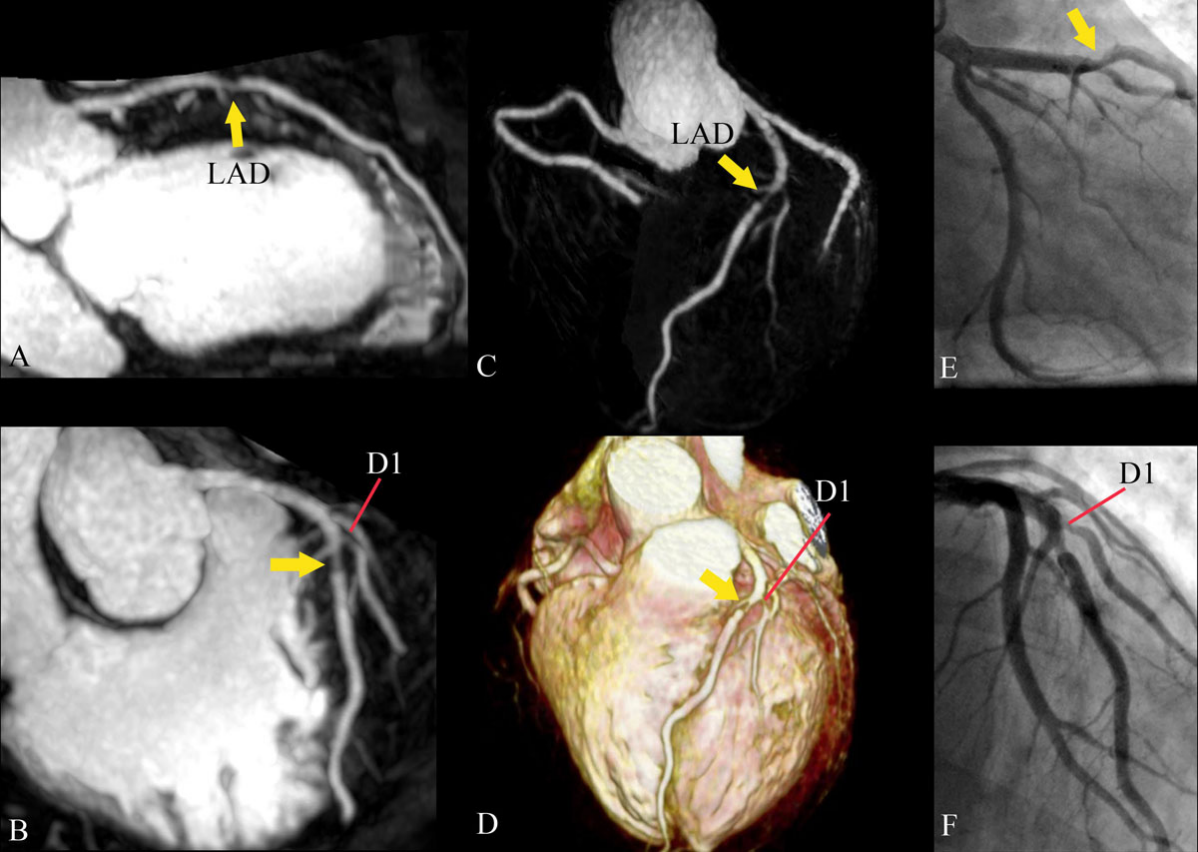
Curved planar reconstruction (CPR) image (A), Sliding thin slab maximum intensity projection (MIP) image (B), MIP image of coronary tree (C), and volume-rendered image (D) detect coronary artery stenoses in the LAD (arrow) and first diagonal branch (arrowhead). Good agreement is observed between coronary MRA and X-ray coronary angiography.

## Conclusions

Among patients who were scheduled to obtain conventional x-ray coronary angiography, we found that coronary MRA at 3.0T demonstrates high accuracy for detection of significant coronary artery stenosis. It warrants greater consideration as a suitable noninvasive method to exclude obstructive CAD.

## Funding

National Basic Research Program 973 (grant no. 2010CB732600) from Ministry of Science and Technology, China; National Natural Science Foundation of China, grant number 30900355; National Institute of Health, grants numbers NIBIB EB002623 and NHLBI HL38698.

